# Tilt in Online Poker: Loss of Control and Gambling Disorder

**DOI:** 10.3390/ijerph17145013

**Published:** 2020-07-13

**Authors:** Axelle Moreau, Émeline Chauchard, Serge Sévigny, Isabelle Giroux

**Affiliations:** 1 École de Psychologie, Université Laval, Pavillon Félix-Antoine-Savard, Québec, QC G1V 0A6Q, Canada; isabelle.giroux@psy.ulaval.ca; 2 Institut Universitaire sur les Dépendances, Montréal, QC H2M 2E8, Canada; 3 Laboratoire de Psychologie des Pays de la Loire, Université de Nantes, 44035 Nantes, France; emeline.chauchard@univ-nantes.fr; 4 Département des Fondements et Pratiques en Éducation, Faculté des Sciences de L’éducation, Pavillon des Sciences de L’éducation, Québec, QC G1V 0A6, Canada; Serge.Sevigny@fse.ulaval.ca

**Keywords:** online gambling, poker, tilt, pathological gambling, model, predictors

## Abstract

Online poker is a form of gambling where an element of skill may influence the outcome of the game. ‘Tilt’ in poker describes an episode during which the player can no longer control their game by rational decisions. It leads to a loss of control over the game, a loss of emotional regulation, higher cognitive distortion, and a loss of money. This phenomenon, experienced by most players, could be the gateway to excessive gambling. The aim of this study was to assess the links between the frequency of tilt episodes, cognitive distortion, anxiety, depression, sensation seeking and excessive online poker gambling. Our sample is composed of 291 online poker players, with a mean age of 33.8 years (SD = 10.6). Participants completed an online self-assessment questionnaire, measuring the frequency of tilt episodes, cognitive distortion, anxiety, depression and impulsivity. The findings indicated that the frequency of tilt episodes and cognitive distortion were the only significant predictors of excessive online gambling (respectively, *r* = 0.49 and *r* = 0.20). Tilt frequency and cognitive distortion were strongly correlated (GRCS, *r* = 0.60), moderate to low correlations were found for tilt and anxiety (HADS, *r* = 0.40), and positive and negative urgency (UPPS, *r* = 0.27). To date, tilt has seldom been studied, and could improve our understanding of online poker gamblers. It could be a new means of identifying at risk gamblers, and thus facilitating preventive measures specifically adapted to this population.

## 1. Introduction

Texas Hold’em Poker is a form of gambling with potential profits, that involves an element of skill and is recognized as addictive [1,2,3,4]. The skill element enables some players to make poker their profession, and encourages some gamblers to continue and try to improve these skills. To play at their highest level, players need to be able to control themselves, their cognitive abilities and their emotions, and must have good concentration faculties [5,6].

‘Tilt’ in poker is an episode during which the player can no longer control their game with rational decisions [7,8,9]. This phenomenon affects emotional and cognitive behaviors and is associated with dissociative experiences. These episodes can be caused by internal (frustration, tiredness) or external events (statistically improbable sudden loss, long series of small losses), which collectively result in a feeling of frustration [7,8,9,10,11]. There are many consequences of these tilt episodes for players. They lead to a loss of control over the game, a loss of emotional regulation, more cognitive distortion (“I’ll win my money back”, “Luck will come”) [8,12,13], negative emotions (sadness, blame) and a loss of money [8,9,10]. In this vein, tilt may be considered as a transitory form of pathological gambling, and a gateway for the development of a more durable problem [7,8,9,14]. Tilt in online poker shares all characteristics of tilt in traditional poker, except for some external manifestations of tilt related to the material or physical environment around the player [7,8,15,16]. Here is an example of an online poker tilt characteristic not shared with traditional poker, since it requires the use of a computer: “I click faster and hit the keyboard harder”. There is a negative link between the severity of tilting and poker playing experience, and a negative link between players’ experience and problem gambling [5,17,18,19]. It also seems that the more skilled a poker player is, the more capable they are of regulating their behavior and emotions [5,20]. Therefore, being more skilled in the game, and having more regulated behavior when tilting, may be protective against problem gambling in online poker.

According to current international studies, 8 to 30% of online poker players have a gambling problem [2,4,21,22,23,24,25,26]. In France, the Observatoire Des Jeux (ODJ—Gambling Observatory) indicates that 14% of poker gamblers meet the criteria of problematic use [27]. The proportion of probable pathological gamblers is also significantly higher for poker players (7.9%) than for all other types of gamblers combined (2.7%) [28]. In France, regarding the rate of problem gamblers, poker comes in 3rd position, after casino games and slot machines [29]. These reports also highlight the characteristics of the population of poker players. When poker players were compared to gamblers not playing poker, poker players reported a higher desire “to win their money back” (i.e., play again to recover their losses) and/or a greater feeling of guilt during the game [27]. These two elements, often mentioned by online problem poker players, are also reported by poker players when they describe the consequences of a tilt episode [8,15,16]. Problem gambling behavior in poker is linked to a lack of emotional and cognitive inhibition [3,7,28,30]. Accordingly, problem gambling behavior in poker has two main characteristics: a lower emotional regulation and a higher cognitive distortion [3,5,7,28,31].

Many studies have identified statistics predictors of excessive gambling, and these include stress, internal attribution, dissociation, boredom tendency, negative emotions, illusion of control, depression, anxiety and impulsivity [2,4,21,23,24,32]. Among these factors, anxiety, depression, impulsivity and sensation seeking have been identified as the strongest predictors of problem gambling in poker [2,3,24,33,34,35]. All types of online poker players are generally characterized as having high sensation seeking scores, even those reporting a recreational practice of poker playing [33,34,35,36]. Impulsivity has been identified as being a predictor of the Internet Gaming Disorder (IGD) [37,38]. In contrast, three recent studies showed that impulsivity is not linked with problem gambling in online poker [2,32,39]. These divergent results were obtained by using different tools, such as: the Barratt Impulsivity Scale (BIS, [24,32,33,40]), The Impulsive Sensation Seeking Scale (ImpSS, [34,36,41]), The Sensation Seeking Scale version V (SSS-V, [35,39,42]), and finally, The Eysenck Impulsiveness Questionnaire (EIQ, [2,43]). This study measures impulsivity in online poker using the UPPS, an impulsive behavior scale that measures five impulsivity components: negative urgency, positive urgency, lack of premeditation, lack of perseverance, and sensation seeking (French version, [44]), as recommended in the literature [21,33,36]. This tool, described as being more precise, will allow a better understanding of the links that impulsivity, sensation seeking, and problem gambling share in online poker.

Several studies have also shown that problem gamblers report more cognitive distortion than players in control, and in particular, “illusion of control”-type distortions [21,45,46,47]. Nevertheless, knowledge on the role of irrational beliefs developed by online poker players remains limited [45,48]. The link between cognitive distortion and problem gambling has often been demonstrated [49]. However, their association with the phenomenon of tilt has yet to be explored.

The overrepresentation of problem gambling in the poker player population shows that this activity induces emotional and cognitive processes specific to risk [14,50]. Poker can therefore be differentiated from other gambling, not only by the features of the game itself, but also by the psychopathological and behavioral characteristics of the players [3,10,27]. Based on current knowledge, our hypothesis is that the frequency of episodes of tilt may be linked to the problematic use of online poker, and to cognitive distortion [7,14]. The present study aims to show these links, their strength and direction. Our first objective will be to verify the presence of relations between problem gambling in online poker, and the frequency of tilt episodes, cognitive distortion, and psychopathological factors, such as anxiety, depression and impulsivity, using group comparisons and Pearson correlations. It is assumed that tilt frequency, irrational beliefs, anxiety and depression will be higher in problem gamblers. Subsequently, our second objective will be to assess the direction and strength of the relations between these different variables using a structural equation model. The second hypothesis of this study is that tilt could be an important predictor of problem gambling in online poker, and that it is closely linked to anxiety, depression, impulsivity and irrational beliefs.

## 2. Method

### 2.1. Participants

Participants had to be at least 18 years old, speak French fluently, have used an online poker account for more than a year, and have played at least once in the last year. In total, 618 participants have taken the online survey, but 326 were removed from the sample. The final sample was composed of 291 online poker players. These were from France (46% of sample, *n* = 133) and Canada (54%, *n* = 157, from the province of Québec), and 92% were male (*n* = 272). In most cases, removed questionnaires were prematurely interrupted (*n* = 266). The mean age of the population was 33.8 years (SD = 10.6), with 72% (*n* = 211) of the sample having an online poker playing frequency higher than once a week (see Table 1 for other sociodemographic variables).

### 2.2. Measures

Participants answered a self-administered online questionnaire. The first part of the survey provided sociodemographic data (gender, age, professional status) and information on the practice of online poker: frequency of playing (“How often do you play poker?”) and length of sessions (“On average, how long do your poker sessions last?”) The second part of the questionnaire consisted of the scales presented below.

### 2.3. The Problem Gambling Severity Index (PGSI), Adapted for Poker

The PGSI is used to measure problem gambling [51]. It comprises nine items rated on a 4-point Likert scale (0 = never, 1 = sometimes, 2 = most of the time, 3 = almost always). A score of 0 indicates non-problematic gambling, and a score equal to, or higher than, 8 indicates a significant gambling problem. We used the revised thresholds suggested by Currie, Hodgins, and Casey [52] for the “low risk” (score of 1–4) and “moderate risk” (score of 5–7) categories. In this study, the players were grouped together to create the “players in control” category, combining all the players with a score of 4 or less. These players experienced no negative consequences on their daily lives. We also adapted the questionnaire to poker, by stating in the instructions, “while playing poker online, during the last 12 months…”. In our study, the Cronbach’s alpha was 0.80 for this scale.

### 2.4. The UPPS, Impulsive Behavior Scale

The UPPS, used in its French short version [44], measures the four facets of impulsivity: urgency (negative and positive), lack of perseverance, lack of premeditation and sensation seeking. It is composed of 20 items, with 12 reversed items (2, 3, 4, 7, 9, 10, 12, 14, 15, 17, 18 and 20), which are assessed with a Likert-type scale with 4 anchor points (1 = Totally in agreement, 2 = Somewhat in agreement, 3 = Somewhat in disagreement, 4 = Completely in disagreement). This analysis resulted in five traits; Negative urgency (tendency to act rashly under extreme negative emotions), Lack of Premeditation (tendency to act without thinking), Lack of Perseverance (inability to remain focused on a task), Sensation Seeking (tendency to seek out novel and thrilling experiences) and Positive Urgency (tendency to act rashly under extreme positive emotions). This scale shows good psychometric qualities with Cronbach alphas between 0.70 and 0.84 for all subscales, showing good internal consistency for the entire scale.

### 2.5. The Hospital Anxiety and Depression Scale (HADS)

This 14-item questionnaire was developed by Zigmond and Snaith [53]. Seven items refer to anxiety symptoms (HADS-A), and seven others assess depressive symptoms (HADS-D). Each item is scored from 0 to 3, so that the total score falls between 0 and 21 for each of the two sections of this questionnaire. Between 8 and 10, there is some doubt about the presence of a depressive or anxious state. Above 10, a depressive or anxious state is confirmed. In our study, the Cronbach’s alpha was 0.76 for the anxiety subscale, and 0.72 for the depression subscale.

### 2.6. Online Poker Tilt Scale

This scale allows for the measurement of the relative frequency of tilt occurrence in online poker. It was developed using semi-directive interviews, and then validated within the French population [15]. Using 17 items, it assesses ten aspects identified as the characteristics of tilt: dissociation, loss of control, attempt to self-control, frustration, alteration of focus, irritability and anger, feeling of sadness, risk taking, desire to gain, and acting out aggressively. These items are measured on a Likert scale from 0 to 4 (never, rarely, sometimes, often, almost every time). In our study, the Cronbach’s alpha was 0.88 for this scale.

### 2.7. The Gambling Related Cognition Scale (GRCS)

We used the French version [54] of the “Gambling Related Cognition Scale (GRCS)” of [55]. This is a 23-item self-report scale with five sub-types of cognitive distortion. The sub-types are: *Gambling-related expectancies*, which assesses the ’hoped-for’; effects of gambling (“Gambling makes me happier”); *illusion of control*, referring to the perception of being able to control the result of a game (“Praying helps me win”); *predictive control*, focusing on the perception of being able to predict the outcome of a game (“Losses when gambling are bound to be followed by a series of wins”); *inability to stop gambling*, assessing the perception of being incapable of resisting the desire to gamble (“My desire to gamble is so overpowering”); *interpretative bias*, which measures the bias of interpretation in favor of continuing gambling (“Relating my winnings to my skill and ability makes me continue gambling”). In our study, the Cronbach’s Alpha was 0.86.

### 2.8. Procedure

We created two online questionnaires using a Lime Survey, one for France and the other for Canada. Links allowing access to the questionnaires were broadcast to different Facebook groups, respectively French, or French Canadian, on poker forums such as “club poker”, “poker académie”, “poker news”, “poker stratégie” or “Princepoker”, as well as by email to students in two participating universities. Data were collected from September 2017 through to May 2018. All participants completed an informed consent form. The Canadian participants could also participate in a draw following their participation to win one of five $100 gift certificates offered as an inducement. This study was approved in Canada (application number: 2017-109 A-1/05-03-2018), and in France (application number: 2017-037), and the Commission Nationale de l’Informatique et des Libertés (CNIL, déclaration StX2149122r).

### 2.9. Data Analysis

The data were analyzed using SPSS Version 25.0 and AMOS version 25 (IBM Corp: Armonk, NY, USA). First, players were sorted into three groups according to their problem gambling score. The statistical means of these groups were compared on each dependent variable using a one-way ANOVA and Tukey’s post hoc test. Then, analyses were conducted on the complete sample. First, Pearson’s correlation coefficient was used to determine whether there were significant correlations between the PGSI score, OPTS score and the psychopathological variables.

**Figure 1:**
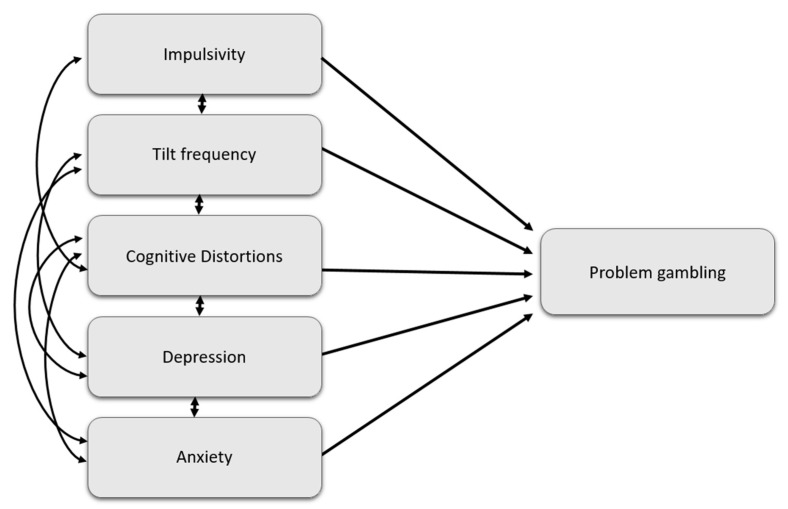
Theoretical-based model predicting problem gambling in the online poker population. For clarity, only the latent variables are shown in the figure.

Then, a structural equation model was performed with AMOS 25, with a congeneric model, in order to test the model and identify the variables that significantly predicted the PGSI score, and to assess their contribution (see Figure 1). Hu and Benthler [56] norms were used to test the model fit: Chi^2^/*df* < 3, *p* > 0.05; “Comparative Fit Index” CFI > 0.95; “Goodness of Fit Index” GFI > 0.95; “Adjusted Goodness of Fit Index” AGFI > 0.80; “Standardized Root Mean square Residual” SRMR < 0.09; the “Root Mean Square Error of Approximation” RMSEA < 0.10. The strength of the relations between the model’s variables was interpreted according to Cohen’s markers [57].

## 3. Results

### 3.1. Descriptive Statistics

Of all the participants, when using Currie, Hodgins, and Casey [52] cut-off scores, 77% (*n* = 224) had a controlled gambling practice, 13.5% (*n* = 39) were classified as moderate risk, and 9.6% (*n* = 28) were probable problem gamblers. The detailed descriptive statistics of the sample are presented in Table 2.

### 3.2. Comparison of Means and Correlations

The means of the scores obtained by the three groups on the five subscales of cognitive distortion, total tilt frequency (OPTS), anxiety, depression and sensation seeking were compared. One-way ANOVA results were significant for all variables, with the exception of age and the following UPPS subscales: positive urgency, lack of premeditation, lack of perseverance and sensation seeking. The Tukey post hoc tests showed that the low risk group was significantly different from the problem gambling group for all the GRCS factors, the OPTS total scale, anxiety, depression and the UPPS subscale negative urgency. Problem gamblers showed significantly higher scores than the other two groups for OPTS variables, and the inability to stop the gambling of the GRCS subscale. The complete results are presented in Table 2.

Concerning the Pearson correlations, our results indicate that the OPTS score correlates significatively with all the other variables, except the premeditation and perseverance subscales of the UPPS. The strongest correlations observed with the OPTS were the inability to stop gambling of GRCS (0.58) and the problem gambling score (0.56) subscales. Concerning the problem gambling score (PGSI), positive correlations with all variables, except the sensation seeking, premeditation and perseverance subscales of the UPPS were observed. Pearson’s correlations for each variable are given in Table 3.

### 3.3. Structural Equation Model

Following the results from the correlation analyses, we have modified the model for a first time, in order to keep the variables with significant correlations with problem gambling only. We tested a structural equation model to examine the problem gambling score, including variables, such as tilt frequency, irrational beliefs, the “emotion based rash action” subscale (positive and negative urgency of the impulsivity scale) and depression and anxiety.

**Figure 2:**
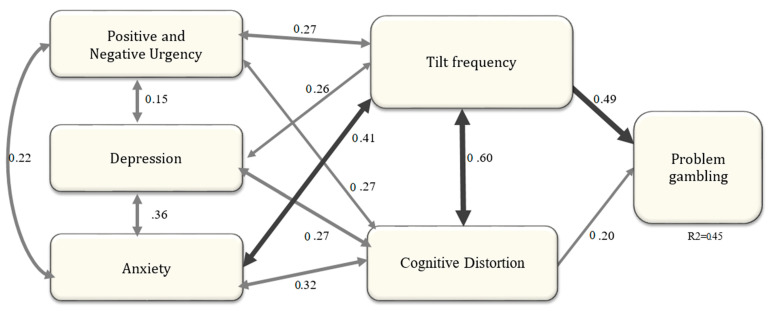
Structural equation model predicting problem gambling in the online poker population (N = 291). For clarity, only the latent variables are shown in the figure.

The data did not fit well with that first model and we made modifications by adding tilt and irrational beliefs as mediators, in order to reach the best fit. The final model indexes were Chi2/df = 1.4, *p* = 0.042, CFI = 0.98, GFI = 0.97, AGFI = 0.94, SRMR = 0.037 and RMSEA = 0.038. These indexes were all very satisfactory, and indicate that the data fits very well with the model. This model indicates that only the tilt frequency (OPTS total) and irrational belief (GRCS total) variables (respectively, 0.49 and 0.20) are significant predictors of problem gambling in online poker, and explain 45% of the score variance. It is also observed that tilt frequency (OPTS total) has strong covariances with irrational beliefs (GRCS, 0.60), as well as anxiety (HADS, 0.40), and a moderate relationship with positive and negative urgency (UPPS, 0.27) and depression (HADS, 0.26) (see Figure 2).

## 4. Discussion

The aim of this study was to examine the links between problem gambling, tilt episodes, cognitive distortion, impulsivity, anxiety and depression, in the specific context of online poker. Our first hypothesis was that tilt frequency, irrational beliefs, anxiety and depression would be higher in problem gamblers. Our second hypothesis proposed that tilt could be an important predictor of problem gambling in online poker, and that it also has close links with the other variables previously identified as risk factors, such as anxiety, depression, impulsivity and irrational beliefs.

The comparison of means indicate that problematic gamblers have higher scores than low-risk gamblers for the frequency of tilt episodes, cognitive distortion, anxiety and depression, but not for impulsivity. Concerning the correlations between the variables, strong links are observed between the variables of tilt, anxiety, depression, cognitive distortion, and two impulsivity subscales (negative and positive urgency). These results confirm the data in the literature, which indicates that anxiety and depression are strong predictors of problem gambling in poker [2,3,4,24,35,39]. However, our results suggest that the frequency of tilt episodes is linked to the problematic use of online poker, and could also be another important risk factor.

Our study is the first to use the UPPS to assess this population, and we found that the scores observed on all the subscales are close to the values obtained by the sample used in the validation study of this tool [44]. Our results indicate a significative difference only in the negative urgency subscale, which is lower for low risk players than for moderate risk players. These results are not congruent with the literature, which indicates that all types of online poker players are generally identified as having high levels of impulsivity and sensation seeking. In the present study, impulsivity does not differentiate problematic players from controlled online poker players. Impulsivity is also not a significant predictor of problem gambling. This result was also found by Biolcati, Passini, and Griffiths [32] who showed that poker players were not more impulsive than the general population. Likewise, the studies by Dufour et al. [2] and Lévesque, Sévigny, Giroux, and Jacques [39] showed that impulsivity is not a predictor of problem gambling in online poker. This result aligns with the characteristics of poker, a game that necessitates high self-regulation capacity, and which does not seem compatible with a very impulsive player profile. On the other hand, the studies showing a significant link are often more centered on sensation seeking than impulsivity. It then seems important for future studies to explore more precisely these concepts and also their links with problem gambling.

Results from structural equation modeling also allow us to validate our second hypothesis. The final model shows that only frequency of tilt and cognitive distortions have a significant direct link with problem gambling. These two factors are also strongly tied to one another. The strongest predictor of problem gambling is tilt frequency. The more frequently that online poker players experience tilt, the more likely they will be to also present a higher problem gambling score. Whilst the links between cognitive distortion and excessive gambling are known [21], the results of this study provide the first empirical support for Browne’s hypothesis [7], that tilt could be a gateway to problematic poker gambling behavior. These results are also consistent with the descriptions of tilt given by players [8,9], who tend to describe that a tilt episode would cause a loss of control and a strong augmentation of irrational beliefs. However, in our model, anxiety and depression are not significant predictors of problem gambling in online poker, but do present moderate covariance links with irrational beliefs and tilt frequency. These results are different from previously published findings, and seem to indicate that, in this population, tilt and irrational beliefs could play a mediating role between anxiety, depression, urgency (positive and negative) and problem gambling. These results are similar to those found by Lévesque, Sévigny, Giroux, and Jacques [39]. Even though there may be no statistically significant direct link with problem gambling, these variables do seem to have an important indirect influence on player behavior, and have previously been identified as ways of detecting, preventing and treating problem gambling in online poker [2,21].

Despite relatively little published research on online poker tilt, this phenomenon needs to be studied more accurately. Whilst a few studies have addressed this issue, none have used a validated tool [7,8,9,10,14,15,19]. Investigating the role of tilt episodes, and more generally episodes of loss of control of playing behavior, whether they are from problem or controlled gamblers, could help to understand the characteristics of this population. In future research, it would be interesting to assess another population sample, for example English-speaking players, in order to confirm these results. Several other issues remain to be explored, such as whether tilt episodes are experienced by the whole population of online poker players, and why only a small proportion of this population eventually develop problem gambling behaviors. Having identified the predictive factors, it is now essential to identify the protective factors, which could help develop more effective prevention programs for problem gambling.

### 4.1. Clinical Implications

Whilst tilt behavior shares many similarities with problem gambling behaviors, the main difference is that tilt is transitory and is an integral part of learning in poker. To become a better performing poker player, it is necessary to learn to identify and manage the occurrence of tilt episodes [5,14,19,20]. More experienced players say that their only defense against tilt is to stop the gambling session [8,9]. Thus, a player with impulsivity, associated with irrational beliefs, anxiety and depression symptoms, will find it more difficult to stop gambling. The player will be more vulnerable if an episode of tilt occurs. If a player is not able to identify or manage the occurrence of tilt episodes, they will vastly increase their likelihood of adopting a risky or even problematic playing behavior [14]. Future studies, specifically longitudinal and clinical ones, would be necessary to confirm these results and explore these hypotheses, but these first findings make us believe that tilt could be a useful concept for clinicians. Tilt is a notion that players know about, and it is an intrinsic part of how they experience poker. Tilt episodes could be a good way to initiate discussions with players on loss of control, emotional regulation, anxiety, and depression linked to gambling. For the same reason, a tilt screening instrument and information on tilt could be essential parts of problem online gambling prevention programs.

### 4.2. Limitations of the Study

This study has several limitations. The first is related to our specific study population. Online poker players were reluctant to take part in this research. For example, we recorded more than 1000 online form initiations, but received only 291 completed questionnaires. Moreover, the generalization of the results is limited to frequent players. As mentioned, the French sample was composed of gamblers playing poker at least once a week. However, the sociodemographic variables of our sample, such as age, gender and professional status, correspond to the values generally observed in this population [4,21,27,35]. Another limitation is related to the tools used. One of the study limitation might be the use of the PGSI, created following the pathological gambling criteria categorized as an impulse disorder in the DSM-IV [58]. However, at this time, this scale brings a consensus among researchers from the field, and is used in many studies to assess problem gambling in sample of poker players. The PGSI allows for international comparison of the results. Also, the PGSI and GRCS were designed for all gambling games, and not specifically for online poker, which contains a significant element of skill that influences the outcome of the game [3,39]. It remains to be established if these questionnaires are totally suitable for online poker players, and if specific measures (e.g. to assess cognitive distortion and problem gambling) need to be created for this population.

## 5. Conclusions

This study indicates that the frequency of tilt episodes is a good predictor of problem gambling in a sample of online poker players. Moreover, these episodes are linked to cognitive distortion, impulsivity, anxiety and depression. This transitory phenomenon, experienced by most online poker players, could be a gateway to, or a manifestation of, problem gambling. Tilt, which has been little studied to date, could improve the understanding of the special characteristics of this at-risk population of gamblers. If these preliminary findings can be confirmed by additional studies, the identification of tilt episodes could be a new means of identifying high-risk gamblers, and lead to the development of prevention strategies specifically addressing tilt in this population.

## Figures and Tables

**Table 1:** Descriptive statistics of our sample of 291 online poker players.

	Numbers	%
Socio-professional category		
Student	55	28.5
Full time employee	180	61.5
Unemployed	24	8
Other	32	11
Online poker playing frequency		
More than once a day	47	16
Once a day	65	22
2 or 3 times a week	99	34
One time a week	35	12
Twice a month	23	8
Less than once a month	20	7
Length of playing sessions		
Less than 30 min	18	6
Between 30 min and 1 h	20	6.5
Between 1 h and 2 h	75	25.5
Between 2 h and 3 h	62	21
Between 3 h and 4 h	40	13.5
More than 4 h	75	26

**Table 2:** Means comparisons of the three groups of gamblers with one-way ANOVA.

	Low Risk (0–4) *n* = 224	Moderate Risk (5–7) *n* = 39	Problematic (>8) *n* = 28	
Variable	Mean (*SD*) [CI]	Mean (*SD*) [CI]	Mean (*SD*) [CI]	*F(2df) (E-Size)*
Age	34.5 (10.8) [33.1–35.9]	32.8 (10.1) [29.5–36.2]	29.6 (8.3) [25.7–33.6]	2.8 (0.02)
OPTS total	15.6 ^a,b^ (7.9) [14.5–16.6]	22.1 ^a,c^ (7.3) [19.6–24.7]	31.4 ^b,c^ (9.7) [28.4–34.4]	54.3 *** (0.27)
GRCS: Gambling-related expectancies	13.0 ^a^ (4.2) [12.4–13.6]	14.7 (4.2) [29.5–36.2]	17.0 ^a^ (4.1) [25.7–33.6]	12.5 *** (0.08)
GRCS: Illusion of control	5.9 ^a^ (3.0) [5.5–6.3]	6.2 (3.0) [5.3–7.2]	7.5 ^a^ (4.9) [5.6–9.4]	3.1 * (0.02)
GRCS: Predictive control	16.0 ^a,b^ (6.0) [15.2–16.8]	18.5 ^a^ (5.6) [16.7–20.3]	20.4 ^b^ (6.2) [18.0–22.8]	8.7 *** (0.06)
GRCS: Inability to stop gambling	9.5 ^a,b^ (4.9) [8.9–10.2]	13.5 ^a,c^ (6.2) [11.5–15.6]	19.8 ^b,c^ (7.1) [17.1–22.6]	50.2 *** (0.26)
GRCS: Interpretative bias	13.9 ^a,b^ (5.1) [13.2–14.6]	16.6 ^a^ (5.5) [14.8–18.4]	19.1 ^b^ (5.0) [17.2–21.1]	15.7 *** (0.10)
GRCS: Mean score	11.7 ^a,b^ (3.4) [11.2–12.1]	13.9 ^a,c^ (3.5) [12.8–15.1]	16.8 ^b,c^ (3.9) [15.3–18.3]	30.7 *** (0.18)
UPPS Negative Urgency	8.0 ^a^ (2.6) [7.7–8.4]	9.2 ^a^ (3.0) [8.4–10.1]	8.7 (2.7) [7.7–9.7]	3.8 * (0.03)
UPPS Positive Urgency	9.5 (2.6) [9.1–9.8]	9.8 (2.7) [9.0–10.6]	9.9 (2.4) [9.0–10.9]	0.5 (0.00)
UPPS Lack of premeditation	6.7 (1.8) [6.4–7.0]	6.4 (1.9) [5.8–7.1]	6.8 (3.1) [6.0–7.5]	0.3 (0.00)
UPPS Lack of perseverance	7.7 (2.4) [7.3–8.0]	7.6 (2.3) [7.0–8.5]	7.8 (3.1) [6.9–8.7]	0.1 (0.00)
UPPS Sensation seeking	10.7 (2.6) [10.4–11.1]	10.7 (2.7) [9.9–11.6]	11.5 (2.4) [10.5–12.5]	1.19 (0.01)
UPPS Mean score	8.5 (1.5) [8.3–8.7]	8.8 (1.7) [8.2–9.3]	9.0 (1.9) [8.2–9.7]	1.37 (0.01)
HADS Anxiety	5.2 ^a^ (3.2) [4.9–5.7]	6.3 (2.7) [5.3–7.3]	7.4 ^a^ (2.8) [6.2–8.6]	6.6 ** (0.04)
HADS Depression	3.4 ^a^ (2.8) [3.1–3.9]	4.1 (2.7) [3.2–5.1]	5.2 ^a^ (4.2) [4.1–6.3]	4.4 ** (0.03)
HADS Mean score	4.4 ^a^ (2.5) [4.1–4.7]	5.2 (2.2) [4.5–5.9]	6.3 ^a^ (3.0) [5.1–7.5]	6.7 ** (0.05)

Note. * *p* < 0.05; ** *p* < 0.01; *** *p* < 0.001; E-size = effect size; CI = 0.95 Confidence interval; Currie, Hodgins, and Casey’s (2012) PGSI cut off; ^a,b,c^ Same superscripts indicate significantly different means. No-superscript indicates non-significant differences (Tukey post hoc test); df = degrees of freedom; The ANOVAs on GRCS, UPPS and HADS mean scores give similar results as the MANOVAs.

**Table 3:** Pearson correlations for the whole population (*n* = 291).

	1	2	3	4	5	6	7	8	9	10	11	12	13
1. OPTS total	1												
2. PGSI	0.56 ***	1											
3. UPPS: Negative Urgency	0.21 ***	0.18 **	1										
4. UPPS: Positive Urgency	0.21 ***	0.12 *	0.65 ***	1									
5. UPPS: Lack of premeditation	0.04	0.02	0.35 ***	0.25 ***	1								
6. UPPS: Lack of perseverance	0.07	0.03	0.04	0.02	0.36 ***	1							
7. UPPS: Sensation seeking	−0.12 *	0.10	0.30 ***	0.43 ***	0.06	−0.15*	1						
8. HADS: Anxiety	0.37 ***	0.30 ***	0.20 **	0.15 **	0.12 *	0.11	0.07	1					
9. HADS: Depression	0.25 ***	0.21 ***	0.16 **	0.04	0.07	0.14 *	−0.07	0.36 ***	1				
10. GRCS: Gambling-related expectancies	0.28 ***	0.33 ***	0.08	0.05	−0.03	−0.05	0.05	0.25 ***	0.18 **	1			
11. GRCS: Illusion of control	0.22 ***	0.21 ***	0.13 *	0.11	−0.03	0.03	0.05	0.21 ***	0.09	0.39 ***	1		
12. GRCS: Predictive control	0.35 ***	0.28 ***	0.17 **	0.15 **	0.09	0.03	0.14 *	0.27 ***	0.20 ***	0.55 ***	0.44 ***	1	
13. GRCS: Inability to stop gambling	0.58 ***	0.55 ***	0.24 ***	0.20 **	0.15 *	0.11	0.14 *	0.32 ***	0.27 ***	0.52 ***	0.30 ***	0.44 ***	1
14. GRCS: Interpretative bias	0.34 ***	0.35 **	0.11	0.10	0.00	0.00	0.00	0.20 **	0.16 **	0.52 ***	0.31 ***	0.51 ***	0.43 ***

* *p* < 0.05 ** *p* < 0.01 *** *p* < 0.001.
